# Pat’s Criticism

**Published:** 1875-06

**Authors:** 


*PAT'S CRITICISM.*

There's a story that's old,
But good if twice told,
Of a doctor of limited skill,
Who cured beast and man
On the "cold-water plan,"
Without the small help of a pill.

On his portal of pine
Hung an elegant sign,
Depicting a beautiful rill,
And a lake, where a sprite,
With apparent delight,
Was sporting in sweet *dishabille.*

Pat McCarthy, one day,
As he sauntered that way,
Stood and gazed at that portal of pine,
When the doctor, with pride,
Stepped up to his side,
Saying, "Pat, how is that for a sign?"

"There's one thing," says Pat,
"Ye 've left out o' that,
Which, be jabers, is quoite a mistake;
It 's trim and it 's nate,
But to make it complate,
Ye shud have a foine bird on the lake."

"Ah, indeed! pray then tell,
To make it look well,
What name do you think it may lack?"
Says Pat, "Of the same
I 've forgotten the name.
But the song that he sings is 'Quack! quack!'"

*Scribner for April.*

